# Domain-specific impairment in metacognitive accuracy following anterior prefrontal lesions

**DOI:** 10.1093/brain/awu221

**Published:** 2014-08-06

**Authors:** Stephen M. Fleming, Jihye Ryu, John G. Golfinos, Karen E. Blackmon

**Affiliations:** 1 Centre for Neural Science, New York University, USA; 2 Department of Experimental Psychology, University of Oxford, UK; 3 Department of Psychology, City University of New York, USA; 4 Department of Neurosurgery, New York University School of Medicine, USA; 5 Department of Neurology, New York University School of Medicine, USA

**Keywords:** neuropsychology, cognitive control, prefrontal cortex, temporal lobe, consciousness

## Abstract

Humans have the capacity to evaluate the success of cognitive processes, known as metacognition. Convergent evidence supports a role for anterior prefrontal cortex in metacognitive judgements of perceptual processes. However, it is unknown whether metacognition is a global phenomenon, with anterior prefrontal cortex supporting metacognition across domains, or whether it relies on domain-specific neural substrates. To address this question, we measured metacognitive accuracy in patients with lesions to anterior prefrontal cortex (*n = *7) in two distinct domains, perception and memory, by assessing the correspondence between objective performance and subjective ratings of performance. Despite performing equivalently to a comparison group with temporal lobe lesions (*n = *11) and healthy controls (*n = *19), patients with lesions to the anterior prefrontal cortex showed a selective deficit in perceptual metacognitive accuracy (meta-*d’/d’*, 95% confidence interval 0.28–0.64). Crucially, however, the anterior prefrontal cortex lesion group’s metacognitive accuracy on an equivalent memory task remained unimpaired (meta-*d’/d’*, 95% confidence interval 0.78–1.29). Metacognitive accuracy in the temporal lobe group was intact in both domains. Our results support a causal role for anterior prefrontal cortex in perceptual metacognition, and indicate that the neural architecture of metacognition, while often considered global and domain-general, comprises domain-specific components that may be differentially affected by neurological insult.

## Abstract (precis)

Convergent evidence supports a role for anterior prefrontal cortex (PFC) in metacognition—the capacity to evaluate cognitive processes—but whether metacognition relies on global or domain-specific substrates is unknown. Fleming *et al*. report that patients with anterior PFC lesions show impaired perceptual metacognition despite intact memory metacognition, supporting a domain-specific account.

## Introduction

Humans have a capacity to evaluate the success of cognitive processes, known as metacognition (Metcalfe, 1996). Impairments in metacognition are found in a range of clinical syndromes, including traumatic brain injury, Alzheimer’s disease, schizophrenia and drug addiction (for reviews see Pannu *et al.*, 2005; Goldstein *et al.*, 2009; David *et al.*, 2012; Cosentino, 2014). In these conditions, impaired awareness of deficits is linked to reduced engagement in remediation treatment, poor adherence to medication and impaired management of functional difficulties (Goldstein *et al.*, 2009; Medley and Powell, 2010; Carretti *et al.*, 2011; Cosentino, 2014). However, despite a clear imperative to understand and ameliorate these deficits, the cognitive architecture supporting metacognition remains poorly understood (Fleming and Dolan, 2012). In particular, while a degree of modularity is accepted for some elements of cognition (Fodor, 1983; Zeki and Bartels, 1998; Pylyshyn, 1999), metacognition and awareness are often considered global phenomena linked to frontal lobe function (Dehaene *et al.*, 1998; Shimamura, 2000). However, this assumption remains untested: is human metacognition domain-general? Or is metacognition itself supported by domain-specific components?

It is possible to quantify metacognitive accuracy in a particular domain by measuring the fidelity of subjects’ trial-by-trial confidence judgements with respect to objective task performance (Clarke *et al.*, 1959; Weiskrantz, 1998; Galvin *et al.*, 2003; Maniscalco and Lau, 2012). Thus, an individual with high metacognitive accuracy is able to accurately recognize and report fluctuations in their performance. Evidence from studies applying convergent methodologies has established a role for lateral anterior prefrontal cortex [PFC; Brodmann area (BA) 10] in metacognitive judgements of perception (Del Cul *et al.*, 2009; Fleming *et al.*, 2010, 2012; Yokoyama *et al.*, 2010; Lau and Rosenthal, 2011; McCurdy *et al.*, 2013). More generally, the literature on anterior PFC function emphasizes roles in abstract reasoning (Bunge *et al.*, 2005; Badre and D'Esposito, 2007) monitoring of internal states (Christoff and Gabrieli, 2000), higher-order aspects of decision-making (Koechlin and Hyafil, 2007; Badre *et al.*, 2012) and attentional control (Burgess *et al.*, 2007; see Ramnani and Owen, 2004 for a review), functions that are often conceptualized as operating independently of a particular domain (although see Wendelken *et al.*, 2012). As anterior PFC receives input from multiple sensory, mnemonic and motor structures (Ramnani and Owen, 2004) and has undergone phylogenetically recent development (Semendeferi *et al.*, 2010), it is plausible that this region supports a domain-general metacognitive ability in humans. This hypothesis is consistent with findings that activation in lateral PFC (BA 10/46) correlates with confidence across both perceptual and mnemonic decisions (Fleck *et al.*, 2006). Furthermore, covariation between measures of metacognitive accuracy across different tasks has lent support to a domain-general account (Veenman *et al.*, 1997; Song *et al.*, 2011; McCurdy *et al.*, 2013).

However, other work indicates independence of metacognitive accuracy across domains (Kelemen *et al.*, 2000; Baird *et al.*, 2013) and differences between the neural substrates supporting metacognition for memory and perception (Baird *et al.*, 2013; McCurdy *et al.*, 2013). Of particular relevance to the current work, a recent study reported that grey matter volume in the anterior PFC of healthy participants predicted individual differences in the accuracy of retrospective confidence judgements in a visual discrimination task, whereas grey matter volume in a neuroanatomically distinct region of medial parietal cortex predicted metacognitive accuracy in a recognition memory task (McCurdy *et al.*, 2013). A prominent view is that metacognitive judgements are inferential in nature, with different heuristic cues affecting judgements in different domains—for example, the accessibility of target-related information for memory confidence (Koriat, 1993) or response speed for perceptual decisions (Baranski and Petrusic, 1998). However, the extent to which domain-specific metacognitive processes can be separated in studies employing correlative techniques is unclear. For example, while a subset of relevant cues may differ, a common underlying cue such as fluency or ease-of-processing may support metacognitive accuracy in both domains (Kelley and Lindsay, 1993; Alter and Oppenheimer, 2009). Lesion experiments, in contrast, are well placed to identify rare cases in which dissociations between domains are observed following damage (Shallice, 1988).

**Figure 1:**
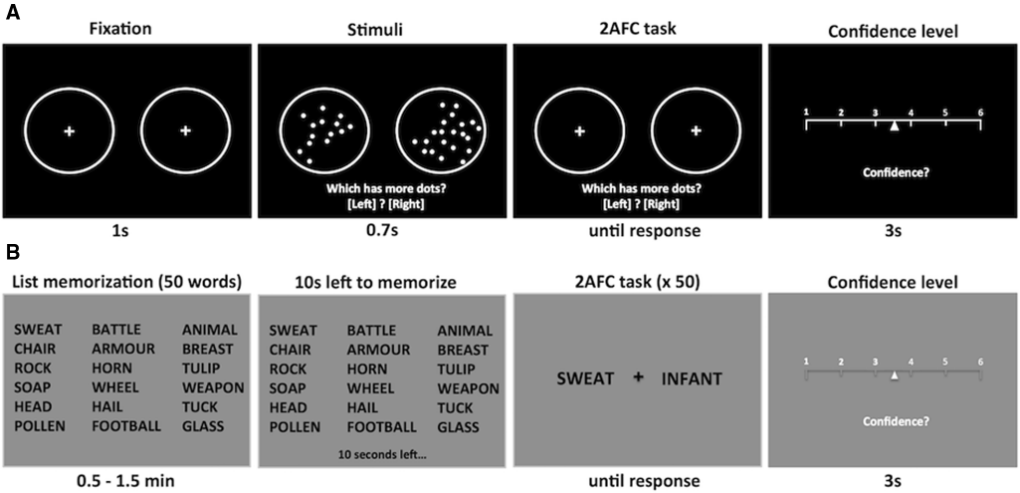
Behavioural tasks. Both the perceptual and memory tasks required two judgements per trial: a two-alternative forced-choice perceptual/mnemonic response followed by an estimate of relative confidence in each decision on a continuous 1-to-6 scale. Task order was counterbalanced across participants. (**A**) Perception task. Participants responded as to which circle (left or right) contained more dots (shown is a schematic representation of the stimulus; actual stimuli each contained ∼ 50 dots) and then rated their confidence in their decision. (**B**) Memory task. Participants studied a list of 50 words arranged in 10 rows and five columns (a six row × three column example is shown here for ease of display). Participants were informed when 10 s remained of the study period. After each study period participants performed a series of two-alternative forced-choice judgements. On each trial two words were presented simultaneously to the left and right of fixation; one word had been presented on the study list and the other had not. Participants were asked to indicate which word (left or right) was on the previously studied list and to subsequently rate their confidence in their decision.

In the current study we used similar visual perceptual and recognition memory tasks to those used by McCurdy *et al.* (2013), and asked whether metacognitive deficits following lesions overlapping with human anterior PFC are domain-specific or domain-general. The perceptual task consisted of a two-alternative forced choice judgement as to which of two briefly presented circles contained a larger number of dots. The memory task consisted of a brief word-list memorization phase followed by a series of two-alternative forced choice recognition judgements. In both tasks participants provided confidence ratings in their decisions on each trial. Given convergent evidence for a role of anterior PFC in perceptual metacognition, we expected metacognitive deficits on the perceptual task in the anterior PFC lesion group. If the contribution of anterior PFC to metacognition is domain-general, we expected the anterior PFC group to additionally show a deficit in a memory task matched for metacognitive demands (Fig. 1).

We studied patients with lesions encompassing the anterior PFC (anterior PFC group), comparing their behaviour with a control group with temporal lobe lesions (temporal lobe group) and a control group of neurologically intact individuals. Our central hypotheses concerned the anterior PFC group, and the selection of temporal lobe patients to form a lesion control group was driven by their relative predominance in the patient database. Previous studies have reported negligible effects of temporal lobe damage on metacognitive accuracy in memory tasks (Prevey *et al.*, 1991; Pannu *et al.*, 2005; Howard *et al.*, 2010, 2013).

In neuropsychological studies of metacognition a key goal is the decoupling of metacognitive accuracy from other changes in primary task performance. Isolating metacognitive accuracy from other confounding factors is problematic as subjective ratings are affected by both task performance and response biases (Galvin *et al.*, 2003; Maniscalco and Lau, 2012). In the present study we use a recently developed signal detection theoretic measure, meta-*d’*/*d’* (Maniscalco and Lau, 2012), to circumvent this problem. Meta-*d’*/*d’* quantifies the efficiency with which confidence ratings discriminate between correct and incorrect trials in each task domain (perception and memory). Using this ratio as a measure of metacognition effectively eliminates performance and response bias confounds typically affecting other measures (Galvin *et al.*, 2003; Masson and Rotello, 2009; Maniscalco and Lau, 2012; Barrett *et al.*, 2013).

## Materials and methods

### Participants

Patients were recruited from the New York University Patient Registry for the Study of Perception, Emotion, and Cognition (NYU PROSPEC). As part of the initial screening for this registry, patients completed a comprehensive battery of neuropsychological tests and a structural MRI scan (T_1_ MPRAGE). Patients were excluded if there was evidence of global cognitive dysfunction on the Full-Scale Intelligence Quotient (FSIQ) from the Wechsler Adult Intelligence Scale-Fourth Edition (i.e. FSIQ < 70) or diffuse atrophy on the MRI scan. Patients with lesions that overlapped with the anterior prefrontal cortex were assigned to the anterior PFC group (*n = *7; two females and five males). A patient control group was formed from patients with anteromesial temporal lobe lesions (*n = *11; six females and five males).

**Figure 2:**
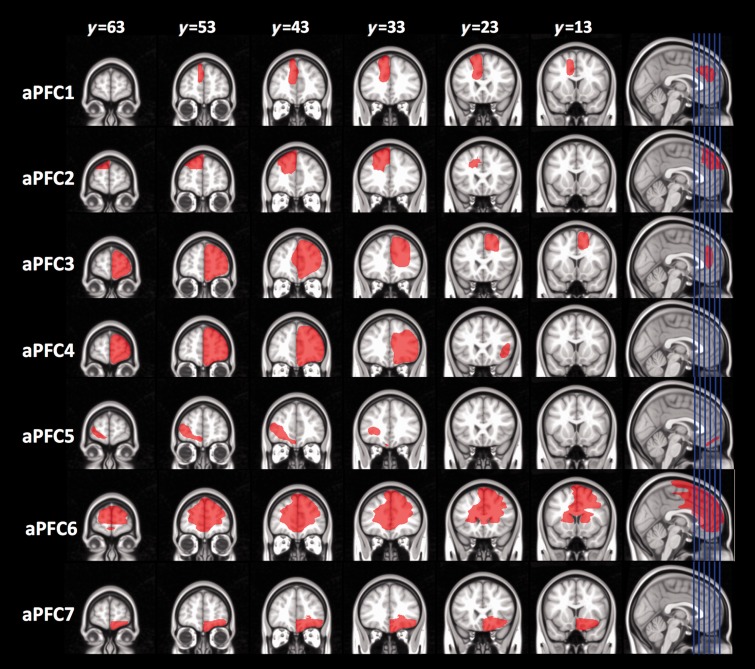
Reconstruction of lesions for each patient in the anterior prefrontal cortex lesion (aPFC) group.

**Figure 3:**
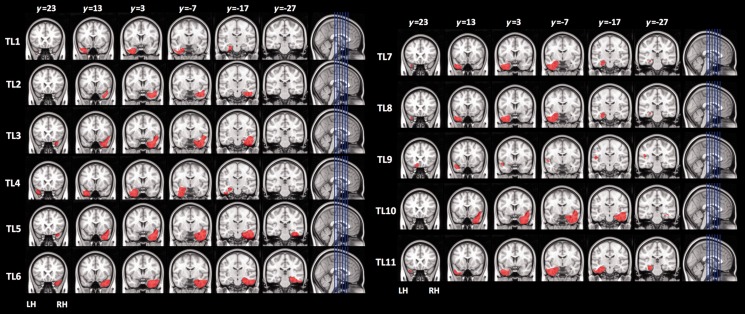
Reconstruction of lesions for each patient in the temporal lobe lesion (TL) group.

Lesion aetiologies for the two patient groups were surgical resection for the treatment of tumours and/or epilepsy (see Figs 2 and 3 for lesion reconstruction maps). In one temporal lobe patient the lesion extended into the basal frontal lobe, posterior to the frontal pole. All patients were tested during the chronic phase of recovery, at least 6 months after surgery.

Healthy control participants (*n = *19; four females and 15 males) were recruited from the community by advertisement. Exclusion criteria included any history of psychiatric disorder, medical disorders affecting the nervous system or current psychotropic/neurologic medications. Healthy control participants were screened in the same manner as patients, including administration of a comprehensive battery of neuropsychological tests and a structural MRI scan (T_1_ MPRAGE). Neuropsychological test scores and MRI scans were unavailable for one healthy control participant. MRI scans from the remaining 18 healthy control participants were reviewed by a board certified neurosurgeon (J.G.G.) to establish the absence of incidental findings. Both patients and control participants had normal or corrected-to-normal vision and hearing.

Healthy controls and patients were administered a neuropsychological test battery assessing cognitive function in the domains of language, visuospatial function, working memory, processing speed, memory, and attention/set-shifting. Core variables from each test were selected to describe and compare performance in the anterior PFC, temporal lobe and healthy control groups. The neuropsychological tests and core variables included: Wechsler Adult Intelligence Scale-IV (WAIS-IV) (FSIQ, Verbal Comprehension Index, Perceptual Reasoning Index, Working Memory Index, and Processing Speed Index; Wechsler, 2008); California Verbal Learning Test-II Delayed Recall (Delis *et al.*, 2002); Trail Making Test B (attention/set-shifting) (Tombaugh, 2004). All raw scores were standardized to the age-matched normal reference group from published manuals (Delis *et al.*, 2002; Tombaugh, 2004; Wechsler, 2008). Although z-scores were generated from normalization to the California Verbal Learning Test-II and Trail Making Test B age-matched reference groups, these were transformed to Standard Scores [mean = 100; standard deviation (SD) = 15] for ease of comparison with the WAIS-IV indices. One-way ANOVAs were used to compare standardized scores across study groups.

Groups did not differ in terms of age [*F*(2,34) = 0.03, *P* = 0.97]. Neuropsychological test scores did not differ across groups (Table 1; all *F* < 1.97; *P* > 0.16), with the exception of verbal recall scores [California Verbal Learning Test-II Delayed Recall; *F*(2,33) = 3.36; *P* = 0.048; Kruskal-Wallis, χ^2^(2) = 6.38, *P* = 0.041] which were significantly lower in the temporal lobe group compared to healthy controls [*t*(27) = −2.5, *P* = 0.02]. There was a trend effect of group on FSIQ [*F*(2,32) = 3.00, *P* = 0.064; Kruskal-Wallis, χ^2^ (2) = 4.42, *P* = 0.11; Table 1] due to lower FSIQ in the two patient groups compared to healthy control subjects [anterior PFC versus healthy control: *t*(22) = 2.10, *P* = 0.047; rank-sum, *P* = 0.046; temporal lobe versus healthy control: *t*(26) = 1.68, *P* = 0.11; rank-sum, *P* = 0.061]. The two patient groups did not differ in FSIQ [*t*(16) = 0.40, *P* = 0.87], time since lesion [*t*(16) = 1.56, *P* = 0.14] or lesion volume [anterior PFC median lesion volume = 49.6 cm^3^; temporal lobe median lesion volume = 44.6 cm^3^; *t*(16) = 1.74, *P* = 0.10]. Neuropsychological data and patient characteristics are summarized in Table 1.

**Table 1:** Summary of patient and control group characteristics

	Anterior PFC group (*n = *7)	Temporal lobe group (*n = *11)	Healthy control group (*n = *19)*
Sex	2 F / 5 M	6 F / 5 M	4 F / 15 M
Handedness	7 R / 0 L	10 R / 1 L	17 R / 2 L
	**Mean (SD)**	**Mean (SD)**	**Mean (SD)**
Age (years)	43.1 (15.8)	43.6 (9.2)	42.3 (16.4)
Time since lesion (years)	2.2 (2.3)	4.3 (3.2)	N/A
Lesion volume (cm^3^)	98.1 (108.6)	41.0 (18.5)	N/A
Full-scale IQ	96 (17)	102 (12)	112 (16)
	**SS mean (SD)**	**SS mean (SD)**	**SS mean (SD)**
Verbal Index (VCI)	105.9 (17.2)	109.1 (12.5)	117.1 (14.3)
Perceptual Reasoning (PRI)	96.1 (15.3)	96.8 (13.7)	105.4 (16.2)
Working Memory (WMI)	95.3 (17.2)	102.5 (13.9)	106.6 (16.7)
Processing Speed (PSI)	92.5 (14.4)	98.9 (10.2)	99.6 (14.1)
Verbal Memory (CVLT-II DR)	84.8 (20.6)	80.1 (18.3)^†^	99 (20.8)
Attention/Set-shifting (TMT-B)	84.4 (25.2)	93.1 (20.4)	95.2 (19.9)

All neuropsychological test scores are transformed to standard scores (mean = 100; SD = 15) for ease of comparison. F = female; M = male; R = right; L = left; SS = standard score; VCI = Verbal Comprehension Index; PRI = Perceptual Reasoning Index; WMI = Working Memory Index; PSI = Processing Speed Index; CVLT-II DR = California Verbal Learning Test-II Delayed Recall; TMT-B = Trail Making Test B.

*Neuropsychological scores were not available for one healthy control participant.

^†^The temporal lobe group had lower scores on CVLT-II DR relative to the healthy control group: t(27) = −2.5; *P* = 0.02.

The experiment was carried out after ethical approval by the local ethics committee (New York University Committee on Activities Involving Human Subjects). All participants gave written informed consent before starting the experiment, and received $20 per hour for their participation.

### Stimuli and procedures

Experiments were programmed in MATLAB (MathWorks) using Psychtoolbox (Brainard, 1997). Participants sat in front of a computer screen at a comfortable viewing distance and completed a perception task and a memory task. For both tasks, participants were asked to make a two-alternative forced choice judgement about what they had perceived or memorized (depending on the task), followed by a confidence rating in their decision (Fig. 1). Task order was counterbalanced between subjects within each group.

The perceptual task consisted of the following sequence of events. Two circles (diameter 5.1°) with small crosshairs in their centres appeared at eccentricities of ± 8.9° for 1 s. The crosshairs then disappeared and a variable number of dots (diameter 0.4°) were displayed inside both circles for 0.7 s. Circles and dots were displayed at maximum contrast (white) on a black background. After stimulus presentation, participants were instructed to guess which circle, left or right, contained more dots. If the left circle contained more dots, the participant was instructed to press the ‘left arrow’ key, whereas if the right circle contained more dots, the ‘right arrow’ key should be pressed. The number of dots within each circle was bounded between 1 and 100. One randomly selected circle always contained 50 dots; the other circle contained a variable number of dots. The difference in dot number (Δ*d*) between the two circles was titrated such that each participant’s performance was maintained at a constant level using a one-up two-down staircase procedure as used previously (Fleming *et al.*, 2010, 2012). After two consecutive correct responses, Δ*d* was decreased by one dot; after one incorrect response, Δ*d* was increased by one dot. The aim of the staircase procedure was to equate the difficulty of the perceptual task between individuals. In total, each participant completed 200 perception trials (eight blocks × 25 trials per block).

The memory task followed the protocol developed by McCurdy *et al.* (2013). Participants were presented with 50 English words simultaneously on the screen and asked to memorize as many as possible for 0.5, 1, or 1.5 min. Words were generated using the Medical Research Council Psycholinguistic Database (Wilson, 1988). Each word was four to eight letters long, had one to three syllables, and had familiarity, concreteness, and imagability ratings between 400 and 700. When there were 10 s left of the study phase participants were notified by a message appearing on the screen below the 50 words presented. Following the study phase, participants completed a series of two-alternative forced choice old/new judgements. Test words appeared either side of a centrally presented crosshair. Participants were instructed that one of the words would be from the previous list (‘old’), while the other would be new. Participants were instructed to choose the word they remembered seeing from the previous list. If the remembered word was on the left side of the crosshair, the participant should press the ‘left arrow’ key and vice versa. There were four blocks of trials which varied by study time. Specifically, each participant completed one block with 0.5 min of study time, two blocks with 1 min of study time, and one block with 1.5 min of study time. The order of study times and word lists was counterbalanced between participants. In total, each participant completed 200 memory trials (four blocks × 50 trials per block).

On each trial in both tasks, participants were presented with a sliding scale to indicate their confidence level in the corresponding decisions they had made. The sliding scale ranged from 1 (low confidence) to 6 (high confidence) and participants were encouraged to use the whole scale. Responses were made by sliding the cursor using the ‘left arrow’ and ‘right arrow’ keys. The scale cursor was initialized at a random location between ratings ‘3’ and ‘4’ on each trial. The confidence scale accepted participants’ input for 3 s, followed by a change in cursor position from white to red to confirm the selected rating (500 ms). Participants received no feedback during the main experiment about their responses.

Before the main task, participants were provided with practice blocks. For the perception task there were three practice phases. In the first phase, example stimuli were shown with text below the circles indicating the number of dots in each circle (e.g. ‘40 versus 60’). In the second phase participants completed a series of dot judgements without confidence ratings. This phase familiarized participants with the task and also began to titrate a subject-specific level of Δ*d* by initiating the staircase procedure outlined above. The last phase consisted of 10 practice trials that simulated the main task such that participants became familiar with indicating their confidence. For the memory task there were six practice trials that simulated the main task (both responses and confidence ratings) without requiring word list memorization.

### Lesion overlap analysis

High resolution T_1_ MPRAGE volumes from each patient were normalized to Montreal Neurological Institute (MNI) standard space using FSL FLIRT (FMRIB's Linear Image Registration Tool; http://fsl.fmrib.ox.ac.uk/fsl/fsl-4.1.9/flirt/) (Jenkinson and Smith, 2001). A two-step registration procedure was implemented: (i) a mask was drawn over the lesion area and any surrounding craniotomy defect to prevent a bias in the transformation caused by the presence of these defects; and (ii) voxels within the mask were given a weight of 0 and ignored during 12° affine transformation of the lesioned brain to the standard MNI 1 mm reference volume. Lesions were then manually traced by a neuropsychologist (K.E.B.) on individual slices of the patient’s brain overlaid on to the standard template, with crosschecking across all three planes. Tracing produced a 3D volume with ‘1’ indicating the presence of the lesion and ‘0’ the presence of normal tissue. These images were smoothed (8 mm Gaussian isotropic) using SPM8 software (www.fil.ion.ucl.ac.uk/spm) for visual display of lesions. Lesion overlap maps were constructed by generating a proportion overlap score at each voxel (lesion/no lesion) ranging from 0 to 1.

**Figure 4:**
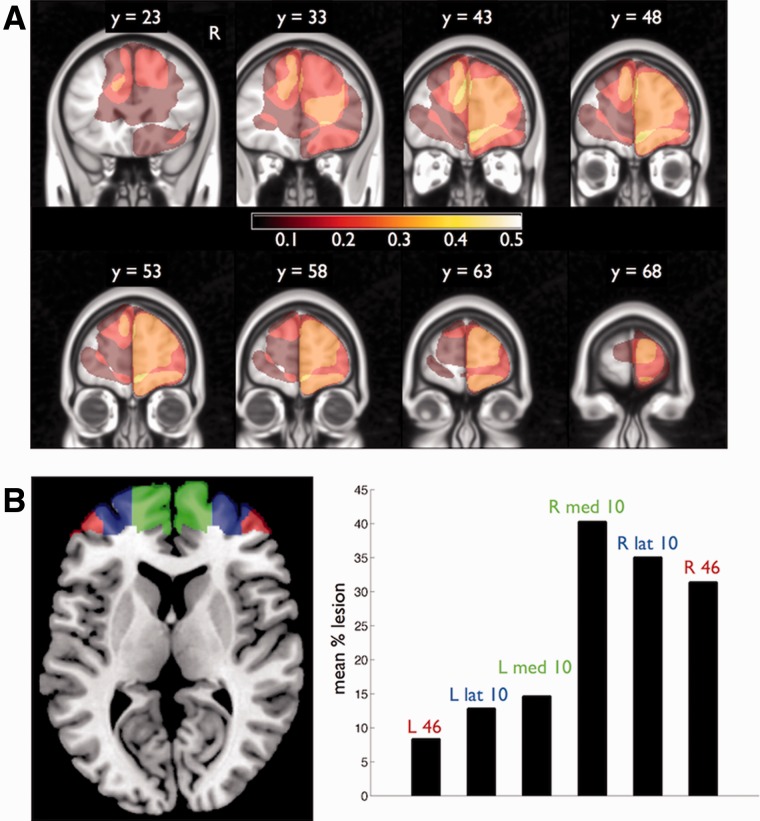
Lesion overlap analysis. (**A**) Coronal slices through an MNI template brain showing overlap of normalized lesion maps for each patient in the anterior prefrontal cortex lesion (anterior PFC) group. Colour bar reflects the proportion of group overlap at each voxel. (**B**) Anterior PFC (BA 10) regions of interest derived from the MRIcron atlas and viewed in axial section (green, blue). All patients in the anterior PFC group had lesions that overlapped with one or both of these regions of interest. Also displayed is neighbouring BA 46 (red). The bar plot shows the mean percentage overlap in each region of interest demonstrating predominant involvement of the right anterior prefrontal cortex.

We further quantified the overlap between lesions and anterior PFC as defined by the boundaries of BA 10 in the MRIcron atlas (http://www.mccauslandcenter.sc.edu/mricro/mricron/), subdivided into lateral (*x* > 20 or *x* < −20) and medial sectors (Gilbert *et al.*, 2006). We additionally computed the overlap with neighbouring BA 46 (Fig. 4). We stress that this assay of lesion encroachment into Brodmann areas is at best probabilistic, as the normalization pipeline and individual differences in sulcal location predict only an approximate correspondence between stereotaxic coordinate and Brodmann area.

### Behavioural data analysis

Performance was quantified as the percentage of correct responses in each task. Performance and confidence in the memory task are additionally reported separately for each time delay (Table 2). The difficulty level (threshold) of the perceptual task was calculated as the mean number of dots added or subtracted to the target stimulus by the staircase procedure (Δ*d*).

**Table 2:** Means and (SD) for metacognitive and performance variables in perception and memory tasks

Task			Anterior PFC group	Temporal lobe group	Healthy control group
**Memory**	**% Correct × study time**	0.5 min	67.7 (11.5)	66.4 (7.7)	68.1 (8.1)
		1 min	70.1 (10.7)	72.8 (9.3)	75.8 (10.3)
		1.5 min	71.7 (11.7)	76.7 (11.6)	76.0 (11.3)
	**Confidence × study time**	0.5 min	3.78 (0.86)	3.53 (0.74)	3.61 (0.91)
		1 min	4.10 (0.90)	4.05 (0.84)	4.03 (0.84)
		1.5 min	4.26 (0.70)	4.31 (0.98)	4.15 (0.90)
	**Meta-*d’/d’***		1.04 (0.38)	0.92 (0.36)	1.09 (0.48)
**Perception**	**% correct**		65.9 (1.8)	65.6 (1.5)	64.6 (2.2)
	Δ***d***		3.70 (1.08)	3.89 (0.95)	3.48 (0.85)
	**Confidence**		3.91 (1.06)	3.83 (0.91)	4.15 (0.51)
	**Meta-*d’/d’***		0.46 (0.28)	0.84 (0.43)	0.88 (0.32)
**Domain-general index**			1.09 (0.80)	0.67 (0.64)	0.38 (0.39)

To estimate metacognitive efficiency we computed meta-*d’* (Maniscalco and Lau, 2012). In a signal detection theory framework meta-*d’* is a measure of type 2 sensitivity (i.e. the degree to which a subject can discriminate correct from incorrect judgements) that is expressed in the same units as type 1 sensitivity (*d’*) (i.e. the degree to which subjects can distinguish stimulus alternatives). This approach dissociates a subject- and domain-specific metacognitive efficiency parameter (meta-*d’/d’*) from both objective task performance and subjective confidence (which both vary on a trial-by-trial basis). Importantly, meta-*d’*/*d’* is a relative measure: given a certain level of processing capacity (*d’*), a meta-*d’*/*d’* value of 1 is metacognitively ideal according to signal detection theory, whereas meta-*d’*/*d’* < 1 indicates that metacognition is lower than expected based on this model. Using this ratio as a measure of metacognitive accuracy effectively eliminates performance and response bias confounds typically affecting other measures (Barrett *et al.*, 2013). Meta-*d’* was fit to each participant’s behavioural data using MATLAB code available at http://www.columbia.edu/∼bsm2105/type2sdt. Before analysis participants’ continuous confidence ratings were binned into four quantiles.

Parametric statistical tests were conducted on log(meta-*d’*/*d’*). A log-transformation weights observations automatically to a ratio scale (Keene, 1995), thus ascribing equal weight to increases and decreases in meta-*d’/d’* relative to a theoretically ideal value of 1. Meta-*d’* is theoretically bounded below by zero, but when fit using an unbounded maximum likelihood estimation procedure estimation error may lead to negative values in practice. Data from one additional healthy control subject were excluded due to a negative perceptual meta-*d’* value precluding calculation of log(meta-*d’*/*d’*).

Where possible, we supplemented results derived from parametric statistics with non-parametric tests (Kruskal-Wallis, rank-sum) and bootstrapped 95% confidence intervals (CI) on summary statistics. We sampled 100 000 bootstrap samples with replacement, and calculated percentiles from the resultant bootstrap distribution (Efron and Tibshirani, 1993). All statistical tests were two-tailed and α was set at 0.05. We tested for a critical group (NC, anterior PFC) × domain (perception, memory) interaction in a linear mixed-model analysis estimated using the *lme4* package in *R*.

### Domain-general index

Previous work indicates that different domain-specific metacognitive accuracies tend to covary in healthy observers (Song *et al.*, 2011; McCurdy *et al.*, 2013; but see Baird *et al.*, 2013). We considered that brain lesions may interfere with this cross-domain consistency. Examining mean metacognitive accuracy scores cannot directly address this question, as it fails to capture the covariance between domains within each group. To address this question, we calculated a domain-general index (DGI) for each subject that quantifies the similarity between their meta-*d’*/*d’* scores in each domain, where $M_{p}$ = perceptual meta-*d’*/*d’* and $M_{m}$ = memory meta-*d’*/*d’*:

$$\text{DGI}=\left|\log M_{p}-\log M_{m}\right|$$

If a subject’s DGI score is low, they have similar scores in both domains (e.g. both high, or both low). We compared DGI scores between groups using independent-samples *t*-tests.

## Results

**Figure 5:**
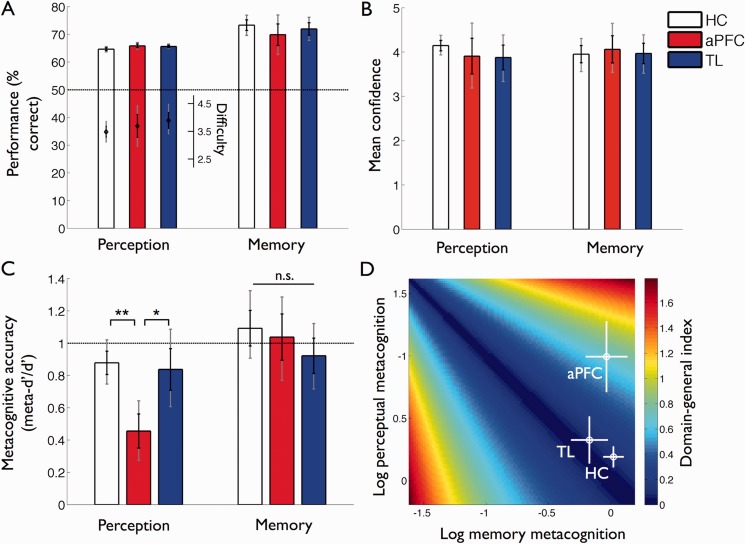
Task performance and metacognitive accuracy. (**A**) Performance (% correct) in each domain for each group (HC = healthy controls; aPFC = anterior PFC lesion group; TL = temporal lobe lesion group). The dashed line indicates chance (50%) performance. The secondary axis shows the average difficulty of the perceptual task (Δd) adjusted online for each participant. Neither performance nor Δ*d* differed across groups. (**B**) Mean confidence in each domain. Average levels of confidence did not differ between groups. (**C**) Metacognitive accuracy scores (meta-*d’*/*d’*) for each domain. The dashed line indicates optimal metacognitive accuracy (meta-*d’/d’* = 1). The anterior PFC group showed a domain-specific impairment in perceptual metacognitive accuracy. (**D**) Illustration of the relationship between domain-specific metacognitive accuracy and the DGI measure reported in the text. Hotter colours reflect a greater DGI score, indicating less consistency across domains. Mean metacognitive accuracy ( ± standard error) for each group is shown for illustration. Note that the group DGI score (Table 2) is affected by both the mean and covariance across domains, whereas only differences between means are apparent here. The anterior PFC group had significantly elevated DGI scores compared to the healthy control group. In **A–C**, black error bars reflect standard errors and grey error bars reflect bootstrapped 95% confidence intervals. **P < 0.01; *P < 0.05; n.s. = not significant.

Experimental data from 37 participants were included in the final analysis. Behavioural measures for each group are summarized in Table 2. There were no significant differences in task performance (% correct) between groups in either domain [Fig. 5A; perception, *F*(2,34) = 1.53, *P* = 0.23; memory, *F*(2,34) = 0.65, *P* = 0.43]. In addition, the average difficulty level of the perceptual task (which was adjusted online for each participant) did not differ between groups [Fig. 5A; *F*(2,34) = 0.70, *P* = 0.51], and each group displayed similar levels of overall confidence in both the memory and perception tasks [Fig. 5B; perception, *F*(2,34) = 0.67, *P* = 0.52; memory, *F*(2,34) = 0.04, *P* = 0.96]. In the memory task greater study time led to significant increases of performance [*F*(2,68) = 13.9, *P* < 0.0001] and confidence [*F*(2,68) = 46.1, *P* < 0.0001] neither of which interacted with group (minimum *P* = 0.45).

Despite equivalent performance and confidence levels across groups, there was a significant effect of group on perceptual metacognitive accuracy [meta-*d’*/*d’*; *F*(2,34) = 5.78, *P* = 0.007; Kruskal-Wallis, χ^2^ (2) = 7.03, *P* = 0.03]. Specifically, the anterior PFC group showed significantly lower perceptual metacognitive accuracy (meta-*d’*/*d’*) relative to both the healthy control and temporal lobe groups [Fig. 5C; anterior PFC < healthy controls: *t*(24) = 3.67, *P* = 0.001, rank-sum, *P* = 0.009; anterior PFC < temporal lobe: *t*(16) = 2.04, *P* = 0.059, rank-sum, *P* = 0.044]. We found no evidence for perceptual metacognitive deficits in the temporal lobe group compared to healthy control subjects [*t*(28) = 0.74, *P* = 0.47]. As meta-*d’*/*d’* quantifies an individual’s metacognitive accuracy in units of task performance (*d’*), it is meaningful to compare this quantity against an optimal metacognition score (meta-*d’*/*d’* = 1). The healthy control or temporal lobe groups did not significantly differ from a metacognitively ideal observer (bootstrap 95% CI), healthy control: 0.75–1.02, temporal lobe: 0.61–1.09; in contrast, the anterior PFC group’s mean perceptual metacognitive accuracy was 46% of optimal (mean = 0.46; bootstrap 95% CI, 0.28–0.64).

Crucially however, the anterior PFC group’s metacognitive accuracy for memory was approximately optimal (mean = 1.04; bootstrap 95% CI, 0.78–1.29) and did not significantly differ from either the temporal lobe [*t*(16) = 0.60, *P* = 0.56; bootstrap 95% CI, 0.91–1.33] or healthy control [*t*(24) = 0.33, *P* = 0.74; bootstrap 95% CI, 0.72–1.12] groups. This dissociation between memory and perceptual metacognition was supported by a significant Group (healthy control, anterior PFC) × Domain (perception, memory) interaction in a linear mixed-effects model (*P* = 0.010). There was no main effect of group on memory metacognitive accuracy [*F*(2,34) = 0.75, *P* = 0.48] and no evidence for memory metacognitive deficits in the temporal lobe group compared to healthy control subjects [*t*(28) = 1.24, *P* = 0.23].

We hypothesized that anterior PFC lesions may disrupt the normal covariation between domain-specific metacognitive accuracies seen in healthy subjects (Song *et al.*, 2011; McCurdy *et al.*, 2013). To test this hypothesis we calculated the absolute difference between perceptual and memory metacognitive accuracies resulting in a DGI for each subject in the study. The lower a subject’s DGI score, the more similar metacognitive accuracies are between the two domains. The anterior PFC group had significantly elevated DGI scores compared to healthy control subjects [*t*(24) = 3.09, *P* = 0.005; rank-sum, *P* = 0.018], but not when compared to the temporal lobe group [*t*(16) = 1.23, *P* = 0.21].

The groups were not balanced for gender ratio or FSIQ (Table 1). In addition, an unavoidable limitation of lesion studies is that patients differ in volume of tissue affected (Figs 2 and 3). To verify that these differences could not account for our results, we performed statistical tests on the influence of these factors on each behavioural measure. Controlling for the influence of FSIQ, gender and lesion volume in analyses of covariance did not affect our conclusions regarding the effect of group membership on meta-*d’*/*d’* (perception, *P* = 0.011; memory, *P* = 0.42), or on the critical Group (healthy control, anterior PFC) × Domain (perception, memory) interaction (*P* = 0.017). No behavioural measure showed significant effects related to either gender or lesion volume (minimum *P* = 0.11). FSIQ was found to correlate positively with per cent correct in the memory task [*r* = 0.51, *F*(1,29) = 13.73, *P* = 0.0009], and negatively with the difficulty parameter of the perceptual task [*r* = −0.44, *F*(1,29) = 6.85, *P* = 0.014], consistent with a contribution of FSIQ to overall task performance. In contrast we did not find effects of FSIQ on metacognitive confidence or meta-*d’*/*d’* (minimum *P* = 0.23).

## Discussion

Here we identify an impairment of perceptual metacognitive accuracy after lesions to the anterior sectors of the prefrontal cortex. The anterior PFC group showed otherwise intact functioning, with equivalent objective performance and mean confidence levels to controls on both the perceptual and memory tasks. Instead, our central finding is of a selective deficit in the trial-by-trial match between confidence and perceptual task performance in the anterior PFC group compared to both healthy controls and a comparison group with temporal lobe damage. Crucially, however, metacognitive accuracy on a memory task matched for metacognitive demands was unimpaired, indicating that the deficit in metacognition in the anterior PFC group is domain-specific.

### Effect of anterior prefrontal cortex lesions on metacognitive accuracy

Previous work applying convergent correlative techniques has identified a link between perceptual metacognitive accuracy and anterior PFC structure and function in healthy individuals. For example, grey matter volume in frontopolar cortex and the integrity of associated white matter correlates with metacognitive ability on a visual discrimination task in neurologically intact individuals (Fleming *et al.*, 2010; McCurdy *et al.*, 2013). A similar correlation has been identified between perceptual metacognitive accuracy and BOLD signal in lateral anterior PFC (Yokoyama *et al.*, 2010; Fleming *et al.*, 2012) and resting-state connectivity between lateral anterior PFC and other brain regions (Baird *et al.*, 2013). However, few studies have permitted a causal manipulation of the role of prefrontal cortex in perceptual metacognition (Rounis *et al.*, 2010).

The current work provides evidence for a causal contribution of anterior prefrontal cortex to perceptual metacognition. In addition, we observed that the anterior PFC group had lowered consistency in metacognitive accuracies across domains. This is consistent with damage to the anterior PFC differentially affecting perceptual metacognition in each subject, thus disrupting the covariation between domain-specific scores seen in healthy controls (McCurdy *et al.*, 2013). We note that patients were studied in the chronic phase of their lesion. Due to the inherent plasticity of brain regions after damage, one possibility is that an initially acute deficit in memory metacognition was compensated by reorganization distant from the lesion site, leading to an apparent dissociation between domains. However such compensation cannot account for the impairment in perceptual metacognition seen in anterior PFC patients, which together with the established contribution of anterior PFC to healthy perceptual metacognition is consistent with a domain-specific deficit triggered by the lesion.

Our study is related but distinct to a previous study that demonstrated decreased subjective reports of visibility in a visual masking paradigm after PFC lesions (Del Cul *et al.*, 2009), with a peak correlation between lesion location and visibility decrease in left anterior PFC. Here we did not find group differences in the average level of perceptual confidence (Fig. 5B). Instead, a trial-by-trial relationship between performance and confidence was attenuated in the anterior PFC patient group. This is consistent with a lack of insight into perceptual task performance following anterior PFC lesions rather than an overall drop in confidence. However our study was not designed to investigate perceptual visibility or conscious access. As perceptual visibility is likely to be dissociable from metacognitive confidence (Charles *et al.*, 2013; Zehetleitner and Rausch, 2013), future studies are needed to determine the respective contributions of PFC to metacognitive accuracy and subjective visibility.

We consider it unlikely that differences in FSIQ between patient and control groups (Table 1) can account for the effects of anterior PFC lesions on metacognition that we observe here. First, controlling for the influence of FSIQ in analyses of covariance did not affect our conclusions regarding the effect of group on measures of metacognition. Second, the anterior PFC and temporal lobe groups were matched for IQ yet still differed in metacognitive accuracy. Finally, we observed no direct influence of FSIQ on metacognitive accuracy, consistent with the absence of a relationship between IQ and metacognition in two previous studies of healthy individuals (Fleming *et al.*, 2012; Weil *et al.*, 2013).

### Contribution of anterior prefrontal cortex to metacognition

What is the role of anterior PFC in perceptual metacognitive accuracy? We have previously proposed that anterior PFC permits an explicit representation of decision confidence (Fleming and Dolan, 2012), in keeping with a hierarchical network architecture for metacognition (Insabato *et al.*, 2010; Pasquali *et al.*, 2010) and the role of anterior PFC in monitoring decision uncertainty (Yoshida and Ishii, 2006; Badre *et al.*, 2012). An alternative, but not mutually exclusive, role for anterior PFC in metacognition is in maintaining stable reference points (criteria) when making confidence judgements. Criteria need to be learnt over time by accruing evidence about the difficulty of the task (Treisman, 1984; Lau, 2007). Indeed, previous studies have identified a contribution of anterior PFC to higher-order aspects of learning (Strange *et al.*, 2001; Daw *et al.*, 2006; Boorman *et al.*, 2011; Kovach *et al.*, 2012).

However, most extant models of metacognitive confidence are silent on the issue of domain, and cannot account for the dissociations observed in the current study. It is possible that parallel hierarchical network architectures are replicated across domains, in keeping with differential connectivity of large-scale brain networks for perceptual and memory metacognition (Baird *et al.*, 2013). On an inferential view of metacognition (Koriat, 1993), if different subsets of heuristic cues contribute to metacognition in different domains, lesions may impair the use of one set of cues to a greater degree than another. Of course, anterior PFC acts in concert with a network of brain regions, including strong interconnectivity with other prefrontal areas (Ramnani and Owen, 2004). Several of our anterior PFC patients had lesions extending into prefrontal white matter (Fig. 2); thus a combination of anterior PFC grey matter loss and reduced connectivity most likely underlies the observed domain-specific deficit in metacognition.

### Effect of temporal lobe lesions on metacognitive accuracy

The temporal lobe lesion group showed impaired delayed recall in the neuropsychological test battery, but similar levels performance and confidence to controls on the recognition memory and perceptual judgement tasks. Previous studies of patients with temporal lobe epilepsy have reported negligible effects of temporal lobe damage on prospective metacognitive judgements of memory such as judgements of learning, showing that patients are able to monitor their memory successfully despite the presence of memory difficulties (Prevey *et al.*, 1991; Pannu *et al.*, 2005; Howard *et al.*, 2010, 2013). In the present study we extend this line of inquiry to document the accuracy of retrospective metacognitive judgements following both perceptual and recognition memory decisions. Metacognitive accuracy in the temporal lobe group was not significantly different from controls in either domain. Together these findings are consistent with relatively intact metacognitive capacities following temporal lobe lesions.

### Neural basis of metamemory

The sparing of metacognitive accuracy for memory in both patient groups raises the question of its neural substrates. Previous lesion studies have implicated the frontal lobe in judgements of memory performance (Janowsky *et al.*, 1989; Vilkki *et al.*, 1998, 1999; Schnyer *et al.*, 2004; Pannu *et al.*, 2005; Modirrousta and Fellows, 2008; for review see Chua *et al.*, 2014). However it is possible that such effects are driven by prefrontal contributions to mnemonic processing itself (Fletcher and Henson, 2001), underscoring the importance of controlling for performance effects when studying memory metacognition. In addition, most studies of meta-memory have focused on prospective judgements of success which may draw on neural substrates distinct to those supporting retrospective confidence judgements (Fleming and Dolan, 2012). The parietal lobe may play an important role in memory metacognition: parietal cortex is regularly activated in functional imaging studies of episodic recall (Wagner *et al.*, 2005) and metamemory (Moritz *et al.*, 2006; Kim and Cabeza, 2007; Chua *et al.*, 2009), and lesions to parietal cortex lead to alterations in retrospective confidence judgements about memory despite performance remaining intact (Simons *et al.*, 2010). It is notable that a recent study demonstrated that memory metacognitive accuracy (meta-*d’/d’*; controlling for task performance) is associated with grey matter volume in the medial parietal cortex in healthy individuals (McCurdy *et al.*, 2013).

An alternative hypothesis is that lateral and medial subregions of the anterior PFC differentially contribute to monitoring of external perceptual and internal mnemonic information, respectively (Baird *et al.*, 2013). Consistent with this notion, examination of anatomical connectivity suggests that external, perceptual information is routed via sensory cortex to the lateral PFC, whereas midline structures such as medial PFC and precuneus are more heavily connected with ‘internal’ information arising in the limbic system (Passingham *et al.*, 2010). In the present study, heterogeneity in lesion location in the anterior PFC group precludes assessment of whether medial or lateral anterior PFC differentially contribute to perceptual metacognitive deficits.

### Limitations

Some limitations of the current work warrant discussion. The size of the anterior PFC group is small (*n = *7), although comparable to other studies examining patients with lesion overlap in anterior PFC (Dreher *et al.*, 2008; Roca *et al.*, 2011; Kovach *et al.*, 2012). Our focus on anterior PFC is strongly motivated by convergent work implicating anterior PFC in perceptual metacognition in healthy observers (Fleming *et al.*, 2010, 2012; Yokoyama *et al.*, 2010; Baird *et al.*, 2013; McCurdy *et al.*, 2013), but localization in the current study should be taken with caution as lesions in the anterior PFC group extended into other areas of PFC (Fig. 4). The decrease in perceptual but not memory metacognition observed in anterior PFC patients is subject to caveats that accompany findings of single dissociations in neuropsychology (Shallice, 1988): if perceptual metacognition is simply more taxing than memory metacognition, it may be that a dissociation between domains would be found despite both tasks relying on the same functional subsystem. We consider this possibility less likely, as tasks were well-matched for metacognitive demands—both required a confidence rating after a two-alternative forced choice judgement—and the meta-*d’* approach takes into account subject-specific differences in primary task performance. In addition, convergent evidence from functional neuroimaging has provided support for separable neural substrates for perceptual and memory metacognition (Baird *et al.*, 2013; McCurdy *et al.*, 2013). However, future work is needed to identify a double dissociation in support of this view.

The perceptual and memory metacognition tasks were chosen to permit comparison with recent structural and functional imaging studies of healthy participants (Baird *et al.*, 2013; McCurdy *et al.*, 2013). However there are differences between the tasks that are potentially orthogonal to the domain in question. For example, our recognition memory task involved verbal stimuli; we cannot rule out the possibility that a different pattern of results would be obtained with memory tasks using non-verbal stimuli such as faces (Vilkki *et al.*, 1999). Indeed, an important goal for future work is the development of perceptual and memory metacognition paradigms that are more closely matched for stimulus characteristics. Similarly it is unclear whether the observed deficit in the anterior PFC group is specific to visual perceptual metacognition, or extends to other perceptual modalities or other aspects of decision-making (De Martino *et al.*, 2013). As few studies have directly compared the neural basis of metacognition across domains (Fleck *et al.*, 2006; Baird *et al.*, 2013; McCurdy *et al.*, 2013) the full space of similarities and differences remains to be explored.

## Conclusion

In sum, our findings reveal that lesions to anterior PFC impaired perceptual metacognitive accuracy while sparing memory metacognitive accuracy. Our findings have both theoretical and practical implications. From a theoretical standpoint, our results are consistent with distributed neural substrates supporting metacognition in different domains, rather than a global mechanism linked to frontal lobe function. On a practical level, metacognition in the clinic is often considered a unitary construct, but our results suggest a more subtle landscape of impaired and intact metacognition. Given the close link between impairments in metacognition and functional outcomes, a greater understanding of the neural and cognitive architecture of metacognition may aid in developing strategies to ameliorate or manage such impairments.

## Abbreviations

DGI: domain-general index
FSIQ: Full-Scale Intelligence Quotient
PFC: prefrontal cortex
